# Left extensive infection in the forearm caused by whitlow infected by mycobacterium tuberculosis: A case report

**DOI:** 10.1097/MD.0000000000008992

**Published:** 2017-12-01

**Authors:** Tao Wang, Gang Zhao, Yong-Jun Rui, Zheng-Feng Lu, Jing-Yi Mi

**Affiliations:** Department of Hand Surgery, Wuxi No. 9 People's Hospital Affiliated to Soochow University, Wuxi, China.

**Keywords:** extensive infection, forearm, mycobacterium tuberculosis, whitlow

## Abstract

**Introduction:**

Whitlow is a common disease in clinic, characterized by pain and swelling of finger. However, few articles had reported on extensive infection in the forearm caused by whitlow infected by mycobacterium tuberculosis (MTB).

**Patient concerns:**

A 70-year-old Chinese female complained of fester in back of left hand for 5 days. She had a history of recurrent whitlow for 14 months and pulmonary tuberculosis (TB). She received treatment in another hospital due to whitlow on July 2016. Then she was treated with incision and drainage. However, whitlow presented again several times before coming to our hospital. She came to our hospital on September 7, 2017 and x-ray of forearm showed that radius, ulna, and carpal were eroded.

**Diagnoses:**

She was diagnosed with left extensive infection in the forearm caused by whitlow infection by MTB.

**Interventions:**

Considering her serious and extensive condition, we performed left forearm amputation on September 12, 2017. We collected some soft tissue cut down during surgery and conducted pathological examination. Finally, pathological result showed MTB infection. Then that patient was treated with antituberculosis therapy.

**Outcomes:**

Up to now, illness condition has not progressed. A recent x-ray of forearm showed no osteolysis in humerus.

**Conclusions:**

Extensive infection in the forearm after recurrent whitlow infection by MTB is rare. So when we face recurrent whitlow, a rapid diagnosis and treatment are required to prevent complications. This case reminds us that recurrent whitlow is dangerous. Attention must be paid to recurrent whitlow. If necessary, amputation should be considered.

## 1 Introduction

Whitlow, often caused by bacteria, is an acute and common infection in hands. Previous study reported the morbidity of whitlow ranged from 2.5% to 15.9%.^[1]^ It was characterized with serious pain and localized erythema. Few whitlow was infected by virus, as some authors reported. Some authors ^[2,3]^ described patient with whitlow caused by herpes simplex virus. And Ramnik et al^[4]^ presented a case of recurrent herpetic whitlow of a 15-month-old child. However, as far as we know, few reports on extensive infected forearm caused by whitlow was infected by mycobacterium tuberculosis (MTB). We report a rare case on extensive infection in the left forearm after recurrent whitlow infected by MTB.

## 2 Consent

The present study was approved by the patient, as well as publication of this case report and any accompanying images, and approved by ethics committee of the Wuxi NO.9 People's Hospital Affiliated to Soochow University.

## 3 Case report

**Figure 1:**
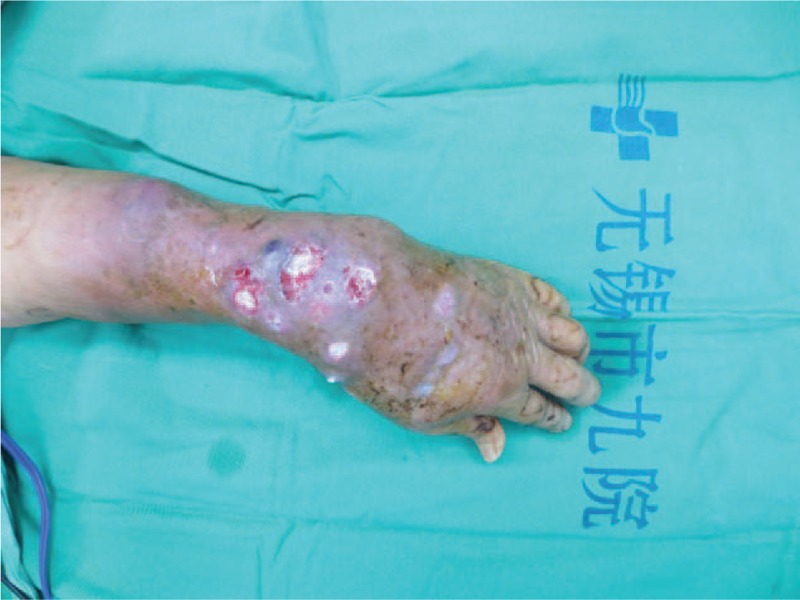
Skin festered in the back of left hand.

**Figure 2:**
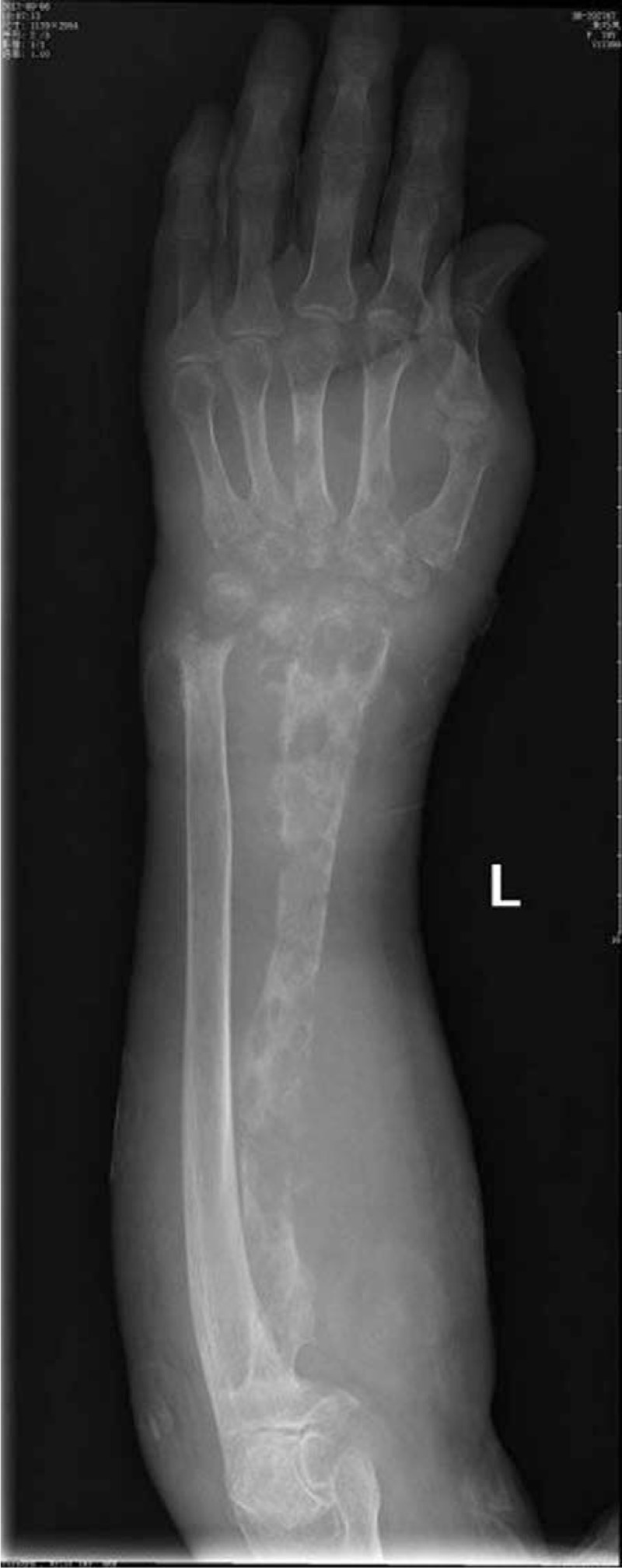
We could see radius, ulna, and carpal were eroded and disappeared in x-ray.

**Figure 3:**
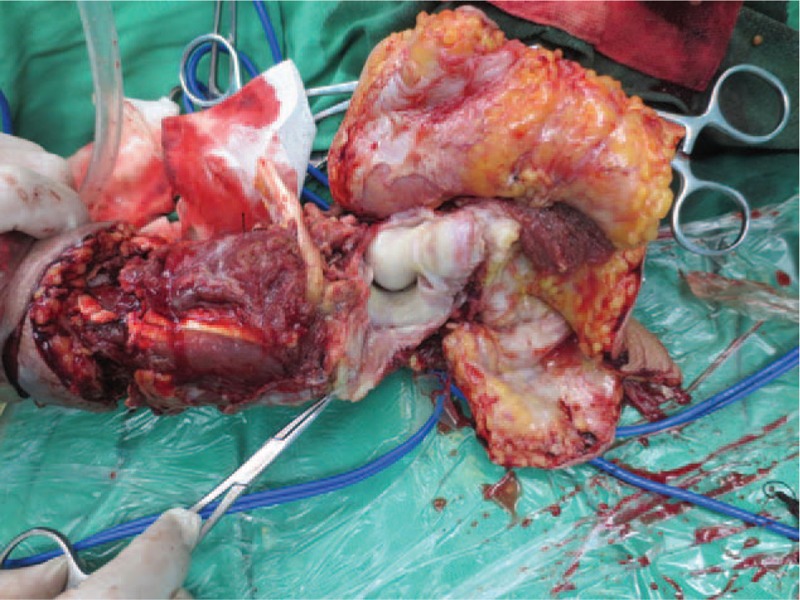
Infection in the soft tissue of left forearm.

**Figure 4:**
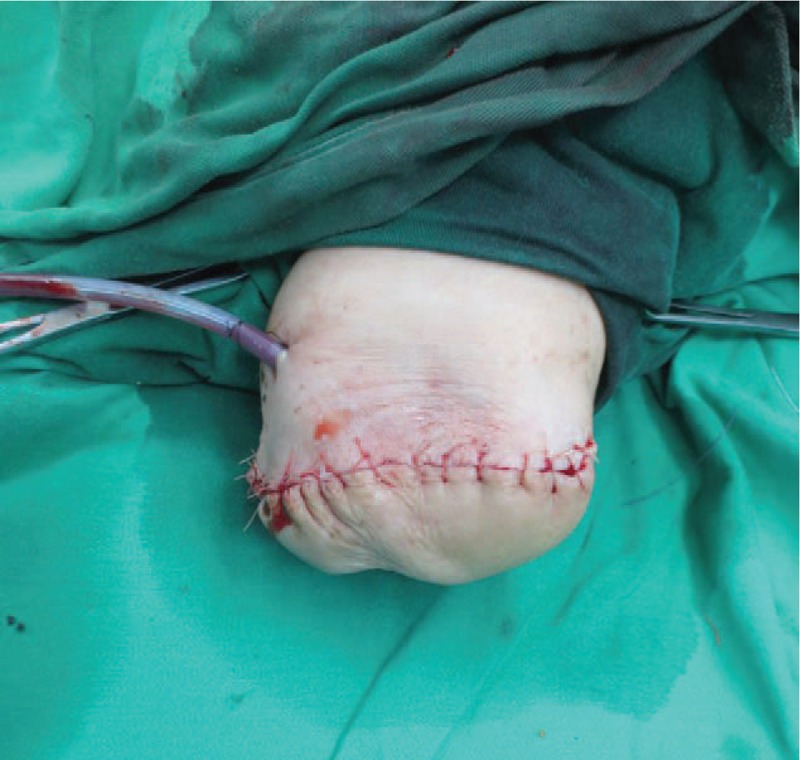
Forearm amputation.

**Figure 5:**
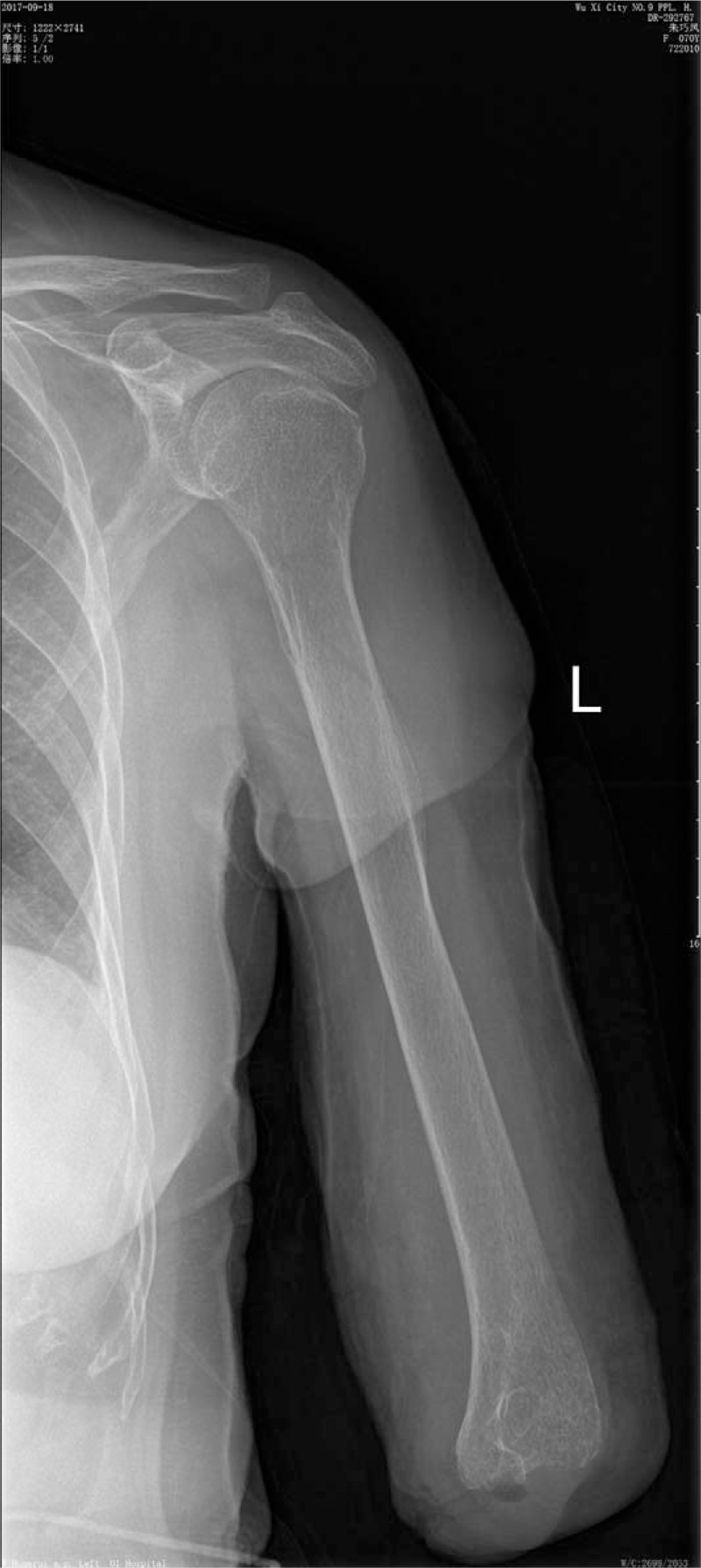
No osteolysis of humerus in x-ray.

A 70-year-old woman complained fester of back of left hand for 5 days. She had a history of recurrent whitlow for 14 months and pulmonary tuberculosis (TB). Whitlow occurred on July 2016 at the first time and she was treated with incision and drainage in another hospital. Then the condition returned to normal. However, the whitlow occurred again and hand relapsed several times before coming to our hospital. The rapid deteriorated condition showed festered skin in the back of left hand on September 2, 2017. On September 7, 2017, she came to our hospital. From Fig. 1, we could see skin festered in the back of left hand and x-ray of left forearm presented radius, ulna and carpal were eroded, as shown in Fig. 2. Body temperature of that patient maintained from 38°C to 39°C. The hemogram on September 8th showed white blood cell (WBC): 11.4 × 10^9^/L, red blood cell (RBC): 4.12 × 10^12^/L, C-reactive protein (CRP): 22 mg/L, erythrocyte sedimentation rate (ESR): 47 mm/h. Considering the serious illness, we decided to performed left forearm amputation. On September 12th, 2017, from Fig. 3, we could clearly see extensive infection in the soft tissue of left forearm. We collected some soft tissue and conducted pathological examination. Finally, pathological result showed MTB infection. That patient was diagnosed with extensive infection in the left forearm caused by recurrent whitlow infected by MTB. We performed left forearm amputation (Fig. 4). That patient was treated with antituberculosis therapy afterwards. One week after surgery, x-ray presented no osteolysis in humerus, as shown in Fig. 5. Laboratory tests revealed WBC: 7.6 × 10^9^/L, RBC: 4.09 × 10^12^/L, CRP: 6 mg/L, ESR: 18 mm/h. Up to now, the condition of that patient is good.

## 4 Discussion

Whitlow is not a rare disease in practice. There are 4 types of whitlow: subcuticular, subcutaneous, thecal abscess, and subperiosteal abscess. The second type, which is perhaps the commonest and the fourth one is relatively least common one. Differential diagnoses include flexor tenosynovitis and paronychia.^[5]^ The similarity in presentation between flexor sheath infection and whitlow may lead to diagnostic confusion and mismanagement. The main treatment to cure this disease is incision and drainage. Milos et al^[3]^ reported the whitlow infected by herpes simplex virus and emphasized the importance of exact diagnosis. Ramnik et al^[4]^ also presented a rare case of 15-month child with recurrent herpetic whitlow. Here, we show a rare case on infected left forearm caused by recurrent whitlow infected by MTB.

Our case presented a 70-year-old woman, who had a history of recurrent whitlow for 14 months and TB, with fester in the back of left hand for 5 days, shown in Fig. 1. The patient had high body temperature and WBC. We could see osteolysis of radius, ulna, and carpal from x-ray of left forearm in Fig. 2. We performed left forearm amputation due to serious and extensive infection in left forearm. Pathological result indicated MTB infection. One week after surgery, body temperature and laboratory tests turned to normal, what's more, there's no osteolysis of humerus in Fig. 5. So far, almost 2 months after surgery, condition of this patient is good, proving that amputation has successfully stopped deterioration of the illness.

We searched for relative studies in PubMed, Embase, the Cochrane library, CNKI, and WANFANG databases considering “whitlow” and “infected forearm” as keywords before surgery, but we failed to find any reports on this topic, meaning that no one could provide experience and reference for us to choose surgical strategy. x-ray of left forearm showed osteolysis just in radius, ulna and carpal, not in humerus. Additionally, in order to prevent development of disease. Considering conditions mentioned above, we thought left forearm amputation as the best plan. Up to now, the progression of illness was effectively curbed, indicating that the efficacy of amputation was satisfactory. This method of treating this disease had some limitations. First, it needs a long follow-up study to prove efficacy; second, this procedure may be radical; third, we need more cases to assess this procedure.

In conclusion, infected forearm due to recurrent whitlow is rare in clinic. As far as we know, few reports had reported on it. We tried new surgical procedure to treat it and had a satisfactory result. We provide a method for surgeons when facing the rare case like this and we need further study to observe efficacy in long-term follow-up.
